# Consensus for the Development of a New Early Warning Score for Predicting Patients' Clinical Deterioration in Angola: A Delphi Study

**DOI:** 10.1155/2024/9070807

**Published:** 2024-09-23

**Authors:** Esmael Tomás, Ana Escoval, Maria Lina Antunes

**Affiliations:** ^1^ Faculty of Medicine of the University Agostinho Neto, Angola–Av. Hoji-Ya-HendaQuintalão do Hospital Américo Boavida, Luanda, Angola; ^2^ NOVA National School of Public Health NOVA University of Lisbon, Portugal–Av. Padre Cruz, Lisboa 1600-560, Portugal; ^3^ Clínica Sagrada Esperança Luanda Angola-Av. Mourtala Mohamed 298 Ilha de Luanda, Luanda, Angola

**Keywords:** Angola, clinical deterioration, consensus, Delphi consensus survey, Early Warning Score, low-income and middle-income countries

## Abstract

**Background:** Nearly 30 years since its inception, the early warning scores (EWSs) remain pivotal, yet variations have emerged for hospital and prehospital use. Aggregated scores, reflecting multiple physiological parameters, outperform single-parameter systems in assessing acute illness severity, though consensus on optimal approaches is lacking. Resource-limited countries, including Angola, lack adapted EWSs, emphasizing the need for cost-effective and adaptable solutions to enhance patient care.

**Objective:** To explore the perspectives of Angolan experts to identify physiological parameters suitable for incorporation into existing EWSs, allowing the development of a new tool adjusted to the healthcare context in Angola.

**Methods:** We conducted a three-round Delphi survey, engaging a national expert panel comprising twenty-five physicians and nurses with expertise in internal medicine, surgery, emergency rooms, intensive care units, and/or teachers at universities or at teaching courses in these fields. Participants were asked to rate items using a five-point Likert scale. Consensus was achieved if the items received a rating ≥ 80% from the panel.

**Results:** Consensus was evident for the inclusion of standard physiological parameters, such as systolic blood pressure, heart rate, respiratory rate, temperature, oxygen saturation, neurological status, and the presence or absence of supplemental oxygen. Furthermore, there was consensus for the consideration of specific items, namely, seizures, jaundice, cyanosis, capillary refill time, and pain—typically not included in the current EWSs. Consensus was reached regarding the exclusion of both oxygen saturation and temperature measurements in healthcare settings where oximeters and thermometers might not be readily available.

**Conclusion:** Angolan experts were able to identify the physiological parameters suitable for incorporation into the basic EWSs. Further study must be conducted to test and validate the impact of the newly suggested vital parameters on the discriminant and predictive capability of a new aggregated model specifically adjusted to the Angolan healthcare setting.

## 1. Introduction

It has been nearly 30 years since Morgan et al. launched the early warning score (EWS), signifying its inception as the pioneering model of its kind [1]. During that time, numerous EWSs have been developed and implemented in various settings to trigger rapid response teams (RRTs) within hospitals and prehospital environments [2, 3]. Aggregated scores of disturbances in multiple physiological parameters have demonstrated a greater robustness as a measure of acute-illness severity compared to single-parameter scoring systems [3]. In addition, these aggregated scores showed significant variation in the physiological parameters taken into consideration [1–3]. There is no consensus on the optimal approach for creating such scores, as the existing models with acceptable predictive accuracy are often specific to the site where the data were collected [4, 5].

Indeed, it is crucial that healthcare professionals attend educational training sessions that enable them to easily identify physiological parameters of deterioration. This knowledge is essential for preventing patients from being at risk of cardiorespiratory arrest and avoidable deaths [3].

Despite the considerable progress in the evolution of EWSs, there persists a shortage of data and recommendations outlining effective approaches in resource-limited low-income and middle-income countries (LMICs) [3, 6]. Angola, after gaining its independence in 1975, endured one of the longest civil wars known in history, leading to significant socioeconomic changes, including changes in terms of income distribution and population demographics. In this context, in most Angolan hospitals and prehospital environments, access to essential resources such as oximeters, thermometers, and sphygmomanometers is often limited. For instance, due to economic constraints and infrastructure shortcomings, the availability of these basic medical instruments may be sporadic or inadequate. In addition, staffing constraints and insufficient professional qualifications further compound the challenges faced by healthcare providers. In many cases, healthcare facilities operate with minimal personnel, who are often overburdened with excessive workloads. As a result, the ability to consistently monitor standard physiological parameters such as oxygen saturation, temperature, and systolic blood pressure is severely hindered. Consequently, healthcare providers in Angola are tasked with managing acute-ill patients with limited access to vital monitoring equipment and facing significant manpower constraints [6, 7].

Currently in Angola, there is presently no EWSs specifically adapted to its reality. Therefore, further studies focusing on EWSs that take into account the local context, cultural sensitivities, and available resources are required. Interestingly, those adjusted EWSs are more effectively aligned with the particular needs and limitations of their community or region. Consequently, they can prevent poor performance and adverse effects on patient care [4, 6].

The aim of the present study was to explore the perspectives of Angolan experts and achieve consensus to identify physiological parameters suitable for incorporation into existing EWSs, allowing for the development of a new tool adapted to the healthcare context in Angola.

## 2. Methods

### 2.1. Study Design: A National Expert Committee Conducted a Delphi Consensus Methodology

#### 2.1.1. Participants

Thirty Angolan experts were identified and formally invited via electronic mail, wherein the author shared hyperlinks to guide them to a form established within the *Jotform*® application. This form comprised inquiries that required considered responses, and twenty-five of the invited experts (83%) accepted the invitation. The selection process of experts partaking in the anonymous voting process of the Delphi survey was meticulously based on their profound knowledge, expertise, and availability to engage in the panel. This diverse group was constituted by physicians and nurses practicing in internal medicine, surgery, emergency rooms, intensive care units, and/or teachers at universities or at teaching courses in these fields.

#### 2.1.2. Delphi Rounds

The Delphi panel involved three rounds, and the following elements were included: anonymity, iteration, controlled feedback, and statistical stability of consensus. A pilot test before all Delphi rounds was conducted to assess any issues. Each round lasted for 1 week [8].

Delphi Round 1: It contained open-ended questions in which participants were asked to identify physiological parameters that they considered important for the creation of a new EWS, specifically suited to the healthcare environment in Angola. The feedback collected during Round 1 served as the basis for formulating the Round 2 survey.

Delphi Round 2: This round included items gathered from Round 1 participants. Five Likert-type questions ranging from 1 to 5 (1: totally disagree; 2: partially disagree; 3: neutral; 4: partially agree; 5: totally agree) were used to assess the importance of each physiological parameter identified as a potential item for developing EWSs in the healthcare environment in Angola. Consistent with prior research, consensus on items was reached if they received a rating of “4: partially agree” or “5: totally agree” from ≥ 80% of the panel. Items that received a rating of 50%–79% from the panel were reintroduced in Round 3 [9].

Delphi Round 3: During this round, participants were presented with responses from Round 2 and were instructed to either “accept,” “reject,” or “rerate” items. The researcher sent an electronic message to each expert with qualitative feedback, informing whether all items were completed according to the instructions. Each participant was informed about the statements that gathered consensus, those that were excluded and those that moved to the next round.

### 2.2. Ethical Considerations

The ethical considerations relevant to the study were observed throughout all stages. The project obtained approval from two distinct local ethics committees (Clínica Sagrada Esperança and Faculty of Medicine of the University Agostinho Neto). To preserve anonymity, all comments added by the panel were not revealed, and more detailed statistical data were not provided. Prior to the initiation of the study, all participants signed an informed consent form.

## 3. Results

### 3.1. Characteristics of the Participants in the Delphi Panel

The characteristics of the participants in the Delphi panel are presented in Table 1, involving 25 experts. The average work experience time was 18.8 years. The majority, constituting 21 (84.0%), were physicians, with 11 (44.0%) specialized in intensive care, and three holding doctorates.

### 3.2. Response Rates

Twenty-five out of 30 invitations were positively responded to, with all 25 participants completing the first survey, yielding an 83% response rate; 24 completed the second survey and 23 completed the final survey. The completion rate for the Delphi study was 92% (calculated as the number of respondents completing all three surveys divided by the number of respondents completing the first survey, expressed as a percentage).

### 3.3. Pilot Test

During the pilot test of the Delphi method, several issues were identified and addressed, including ambiguities in question wording, technical difficulties with the platform, concerns regarding question length, and challenges in understanding instructions. In response to this feedback, researchers refined the questionnaires to improve clarity, relevance, and ease of use for the panel.

### 3.4. Delphi Round 1

The consensus suggests that the following standard physiological parameters, routinely recorded in hospitalized patients—such as systolic blood pressure, heart rate, respiratory rate, temperature, oxygen saturation, neurological status, and the presence or absence of supplemental oxygen—are deemed crucial for the development of new EWSs in the healthcare environment in Angola. However, faced with challenges related to the use of two oxygen saturation scales in certain EWSs, the panel has suggested the adoption of a single oxygen saturation scale. Furthermore, there is deliberation about the potential exclusion of both oxygen saturation and temperature measurements in resource-limited environments where access to oximeters and thermometers may be limited or absent.

In addition, the panel identified eight new items as crucial physiological parameters for inclusion: urinary output, seizures, jaundice, vomiting, abdominal pain, cyanosis, capillary refill time, and pain.

### 3.5. Delphi Round 2

The responses from the panel regarding the significance of each newly recognized physiological parameter as a potential component for constructing EWSs in the healthcare setting in Angola are presented in Table 2. Among them, unanimous agreement was reached in this round for seizures, with an 83% consensus rate. Conversely, vomiting (42%) and abdominal pain (46%) were excluded based on the consensus.

Furthermore, regarding the exclusion of both oxygen saturation and temperature measurements, the panel opposes excluding these elements, underscoring that they should be preserved whenever practical for measurement. However, in healthcare settings where oximeters and thermometers might not be readily available, it is crucial to contemplate an EWS without those components.

### 3.6. Delphi Round 3

The items urinary output, jaundice, cyanosis, capillary refill time, and pain, which received a “4: partially agree” or “5: totally agree” from 50% to 79% of the panel in Round 2, were reintroduced for additional evaluation. A unanimous consensus (≥ 80%) was subsequently reached to exclude urinary output and to include the remaining 4 items.

## 4. Discussion

This study explored the perspectives of Angolan experts from diverse backgrounds to identify physiological parameters suitable for incorporation into existing EWSs, facilitating the creation of a new tool adjusted to the healthcare landscape in Angola.

In alignment with the existing literature, this study revealed a consensus (≥ 80%) for the inclusion of standard physiological parameters, encompassing systolic blood pressure, heart rate, respiratory rate, temperature, oxygen saturation, neurological status (assessed through the AVPU scale—alert, confusion, verbal, pain, and unresponsive), and the presence or absence of supplemental oxygen [1–3, 10]. In addition, consensus was observed for the consideration of specific items, including seizures, jaundice, cyanosis, capillary refill time, and pain reflecting the unique considerations of the Angolan healthcare context.

The importance of reducing the number of physiological parameters in EWSs systems has been investigated, as an extensive set of variables may impact the data collection workload, adding complexity for healthcare professionals in their work and potentially increasing the risk of errors [11, 12]. DeVita et al. studied the inclusion of temperature as a predictor of death in the NEWS model and found no significant effect. Its exclusion resulted in outcomes similar to those obtained with the original model [5, 11]. Therefore, in healthcare settings such as many Angolan hospitals where thermometers might not be readily available, it could be considered consistent to contemplate an EWS without including temperature as one of its components.

While systolic blood pressure is consistently presented and considered crucial for detecting clinical deterioration in patients, a study by Luís et al. demonstrated that a simplified EWS model, which excludes systolic blood pressure, exhibits similar sensitivity and specificity to the full model. This finding suggests that the simplified EWS can effectively identify patients at risk without the need for systolic blood pressure measurements, and proposed a tool to support clinical decision-making in the Portuguese healthcare context, emphasizing the use of fewer data points for a more efficient allocation of health professionals' worktime without compromising its discriminant capacity [11, 12].

Sebat et al. analyzed the value of capillary refill time, a quick, easily performed, and important component of their EWS in identifying patients with a higher likelihood of clinical decline. They provided proof of concept regarding the value of capillary refill time in RRT patients and proposed to undertake further trials to better define its role as part of the bedside assessment of adult hospitalized patients [13].

Pain has been advocated as the fifth vital sign. Purser, Warfield, and Richardson conducted a three-stage audit of pain assessment in a large teaching hospital in the northwest of England. Their study revealed that enhancing the visibility of pain assessment on the patient observation chart led to improved uptake of pain assessment. In addition, identification of high pain scores prompted the implementation of pain management strategies [14].

The consensus to include seizures as an indicator of clinical deterioration and to incorporate it into the EWSs has already been established in numerous studies of the paediatric population. These studies have identified seizures as a risk factor significantly associated with poor outcomes [15].

In summary, the research has concluded against including specific multiple physiological parameters as part of the scoring system for the EWSs. This decision is grounded in the principle that some of these parameters may not always be available during the initial assessment. Their exclusion, however, does not imply their insignificance or suggest that they should not be recorded and considered in the overall clinical evaluation of the patient. Nevertheless, additional research is considered necessary to validate their potential inclusion in the scoring system [5, 11, 12].

Integrating the findings from this study, healthcare practitioners in Angola can now embark on the development and implementation of an EWS system adapted to the unique challenges and resources available within the Angolan healthcare landscape. In addition, the inclusion of specific parameters, namely, seizures, jaundice, cyanosis, capillary refill time, and pain, underscores the importance of addressing context-specific considerations in healthcare decision-making. Ultimately, the creation of an EWS adapted to the Angolan context has the potential to enhance patient outcomes by facilitating timely interventions and optimizing resource allocation.

Moreover, this study lays the groundwork for future research endeavors aimed at validating the proposed parameters and evaluating their impact on the discriminant and predictive capabilities of the EWS model. By refining and validating this model, healthcare practitioners can further refine their ability to deliver effective patient care in resource-constrained environments such as Angola.

## Figures and Tables

**Table 1:** Characteristics of the 25 participants in the Delphi panel.

**Characteristics**	**N = 25**
Mean age (range) (years)	44.8 (33–63)

Mean work experience time (range) (years)	18.8 (6–36)

Academic degrees, *n* (%)	Graduated: 21 (84.0) Master's degree: 1 (4.0) Doctor of Philosophy: 3 (12.0)

Profession, *n* (%)	Physicians: 21 (84.0) Nurses: 4 (16.0)

Specialty, *n* (%)	Intensive care: 11 (44.0) Internal medicine: 6 (24.0) Emergency medicine: 4 (14.0) Surgery: 3 (12.0) Nursing supervision: 1 (4.0)

**Table 2:** Panel responses on the importance of each new physiological parameter identified as a potential item for developing EWSs.

	**Urinary output**	**Seizures**	**Jaundice**	**Vomiting**	**Abdominal pain**	**Cyanosis**	**Capillary refill time**	**Pain**
Totally disagree	1	3	6	7	7	3	2	4
Partially disagree	4	1	0	4	3	2	2	2
Neutral	2	0	3	3	3	1	1	2
Partially agree	6	4	3	3	5	3	3	4
Totally agree	11	16	12	7	6	15	16	12
Partially or totally agree	17 (71%)	20 (83%)	15 (62%)	10 (42%)	11 (46%)	18 (75%)	19 (79%)	16 (67%)

## Data Availability

The underlying data supporting the results of our study are available and accessible. The authors possess the dataset utilized in this research. If there are any inquiries or requests regarding the data, please do not hesitate to contact the corresponding author for further details.
